# Association between Postnatal Dexamethasone for Treatment of Bronchopulmonary Dysplasia and Brain Volumes at Adolescence in Infants Born Very Preterm

**DOI:** 10.1016/j.jpeds.2013.10.083

**Published:** 2014-04

**Authors:** Jeanie L.Y. Cheong, Alice C. Burnett, Katherine J. Lee, Gehan Roberts, Deanne K. Thompson, Stephen J. Wood, Alan Connelly, Peter J. Anderson, Lex W. Doyle, Victorian Infant Collaborative Study Group

**Affiliations:** 1 Neonatal Services, Royal Women's Hospital, Melbourne, Australia; 2 Victorian Infant Brain Studies, Murdoch Childrens Research Institute, Melbourne, Australia; 3 Department of Obstetrics and Gynecology, University of Melbourne, Melbourne, Australia; 4 Department of Pediatrics, University of Melbourne, Melbourne, Australia; 5 Clinical Epidemiology and Biostatistics, Murdoch Childrens Research Institute, Melbourne, Australia; 6 Royal Children's Hospital, Melbourne, Australia; 7 Developmental Imaging, Murdoch Childrens Research Institute, Melbourne, Australia; 8 Florey Institute of Neuroscience and Mental Health, University of Melbourne, Melbourne, Australia; 9 School of Psychology, University of Birmingham, Edgbaston, United Kingdom

**Keywords:** BPD, Bronchopulmonary dysplasia, MRI, Magnetic resonance imaging, PCS, Postnatal corticosteroid

## Abstract

**Objectives:**

To compare brain volumes in adolescents who were born extremely preterm (<28 weeks gestation) who had received postnatal dexamethasone, and to determine if there was a postnatal dexamethasone dose–response effect on brain volumes.

**Study design:**

Geographical cohort study of extremely preterm adolescents born in 1991-1992 in Victoria, Australia. T1-weighted magnetic resonance imaging was performed at 18 years of age. Segmented and parcellated brain volumes were calculated using an automated segmentation method (FreeSurfer) and compared between groups, with and without adjustment for potential confounders. The relationships between total postnatal dexamethasone dose and brain volumes were explored using linear regression.

**Results:**

Of the 148 extremely preterm participants, 55 (37%) had received postnatal dexamethasone, with a cumulative mean dose of 7.7 mg/kg. Compared with participants who did not receive postnatal dexamethasone, those who did had smaller total brain tissue volumes (mean difference −3.6%, 95% CI [−7.0%, −0.3%], *P* value = .04) and smaller white matter, thalami, and basal ganglia volumes (all *P* < .05). There was a trend of smaller total brain and white matter volumes with increasing dose of postnatal dexamethasone (regression coefficient −7.7 [95% CI −16.2, 0.8] and −3.2 [−6.6, 0.2], respectively).

**Conclusions:**

Extremely preterm adolescents who received postnatal dexamethasone in the newborn period had smaller total brain tissue volumes than those who did not receive postnatal dexamethasone, particularly white matter, thalami, and basal ganglia. Vulnerability of brain tissues or structures associated with postnatal dexamethasone varies by structure and persists into adolescence.

See editorial, p 687

Postnatal corticosteroids (PCS) to prevent or treat bronchopulmonary dysplasia (BPD) in preterm infants have beneficial effects, such as earlier extubation and lower rates of BPD.^1,2^ However, PCS use, especially dexamethasone, has also been associated with adverse neurodevelopmental outcomes, in particular cerebral palsy.^1^

Experimental models have shown the vulnerability of the developing brain to corticosteroids, including exacerbation of neuronal and astroglial injury in the hippocampus,^3,4^ and impaired neurogenesis, myelination, and brain cell division.^5,6^ There is recent evidence suggesting that the newborn cerebellum may be at greater risk than other structures in the brain given that it has the highest number of glucocorticoid receptors in the brain, localized to the external granular layer.^7^

Magnetic resonance imaging (MRI) allows the characterization of brain alterations associated with preterm birth. Several studies in the last decade have reported MRI-derived brain structural alterations associated with PCS, particularly in total brain tissue, cortical gray matter, subcortical gray matter, hippocampus, and cerebellum, which may relate to the neurodevelopmental deficits reported in preterm children.^8-14^ The corticosteroids used in these previous MRI studies were mainly dexamethasone and hydrocortisone, and MRI performed at term-equivalent age, with the exception of 1 study where MRI was performed at 8 years of age.^11^ These small studies have not been able to conclusively establish either a dose–response relationship or regional vulnerability within either gray or white matter to corticosteroids. More importantly, there are no studies of alterations of long-term brain volume in adolescence following PCS use. We sought to address these research gaps in a large prospective longitudinal cohort of extremely preterm (<28 weeks) survivors who had a brain MRI at 18 years of age.

Specifically, the aims of this study were to compare the volumes of multiple brain tissues and structures between extremely preterm adolescents who had received postnatal dexamethasone in the newborn period with those who did not, and to determine whether there is a dose–response relationship of postnatal dexamethasone with brain volumes at age 18 years. We also aimed a priori to explore regional vulnerability within cortical gray and white matter if a group difference was found in either of these brain tissues in the initial analysis.

We hypothesized that extremely preterm adolescents who had received postnatal dexamethasone would have smaller total brain tissue volumes compared with those who had not, as well as smaller volumes in cortical gray matter, basal ganglia and thalami, cerebellum, and hippocampus as previously reported at term-equivalent age. We expected there would be no regional vulnerability within either cortical gray or white matter. We also hypothesized that higher cumulative postnatal dexamethasone doses received in the newborn period would be associated with smaller brain volumes at age 18 years.

## Methods

Participants of this study were part of a geographic, prospective cohort of all extremely preterm infants born in the state of Victoria, Australia, between January 1991 and December 1992 enrolled at birth. Study participants had been previously assessed at ages 2, 5, and 8 years.^15-18^ All participants were invited to a health and developmental follow-up at age 18 years, which included an MRI scan.

Ethical approval for the original and follow-up studies was from the Human Research Ethics Committees of the Royal Women's Hospital, Mercy Hospital for Women, Monash Medical Centre, and Royal Children's Hospital, Melbourne. Written consent was from all participants and their parents if they were younger than 18 years of age at the time of assessment.

Perinatal and neonatal data were collected prospectively from medical records. Major brain injury (grade 3 or 4 intraventricular hemorrhage or cystic periventricular leukomalacia) was diagnosed by serial cranial ultrasound during the primary hospitalization. BPD was diagnosed in infants with respiratory distress who were oxygen-dependent at 36 weeks corrected gestational age. Corticosteroids to treat or prevent BPD were prescribed at the discretion of the treating clinicians in each of the 4 tertiary level nurseries in the state of Victoria. The primary corticosteroid prescribed was dexamethasone, either parenteral or oral, started after the first week of life. Occasionally, doses of hydrocortisone were given to infants if they were considered to be at increased risk of adrenal failure during a time of crisis, such as surgery. As hydrocortisone use was very uncommon, doses of hydrocortisone were converted to dexamethasone equivalent doses (assuming relative potencies of dexamethasone 25 and hydrocortisone 1) and we refer to postnatal dexamethasone throughout the manuscript, which includes hydrocortisone. Data on postnatal dexamethasone use were collected throughout the complete neonatal stay in the neonatal nurseries. The total dose in mg/kg was recorded.

### MRI

MRI was performed at 2 of the study sites, each using a Siemens 3T MAGNETOM Trio MRI system (Siemens, Erlangen, Germany), a 12-channel receive-only head coil, and the same acquisition protocol. For this study, three-dimensional T1-weighted Magnetization Prepared Rapid Gradient Echo datasets were obtained using the following measurements: non-isotropic voxels 0.7 × 0.7 × 1.2 mm, field of view 230 mm, repetition time 1800 ms, echo time 2.67 ms, and flip angle 9°.

### Image Analysis

MRI data were processed by a single operator, blinded to group status, on Linux workstations using the automated FreeSurfer image processing suite (stable release v. 5.0, http://surfer.nmr.mgh.harvard.edu).^19,20^ Images were excluded if they were considered of unacceptably poor quality because of artifact and those with significant structural abnormalities that precluded FreeSurfer registration. The magnetic resonance images were visually inspected and manually edited as required during the automated processing pipeline.

The automated labeling system for whole brain segmentation within FreeSurfer was used and volumes were estimated for cortical gray matter, white matter, thalamus, caudate, putamen, pallidum, hippocampus, amygdala, cerebellar white and gray matter, and intracranial volume (including the ventricular volumes).^20^ The total volumes for caudate, putamen, and pallidum were presented individually and also combined to represent basal ganglia volume. Cerebellar volume was taken as the sum of cerebellar white and gray matter. Total brain tissue volume was the combined volumes of all the above brain structures including the brainstem (ie, excluding cerebrospinal fluid volume). Freesurfer's 34 parcellated cortical gray matter regions using the Desikan atlas^21,22^ were combined to form 20 neuroanatomical regions (Table I; available at www.jpeds.com). Freesurfer estimates the volume for each brain tissue or structure separately for each hemisphere with the exception of intracranial volume, but for our analyses we combined volumes from both hemispheres.

### Statistical Analyses

Data were analyzed using STATA 12.0 (StatCorp, Houston, Texas). Participant characteristics were compared between participants with and without analyzable MRI, using *t* tests or χ^2^ analyses. Group differences in brain volumes were explored using linear regression fitted using generalized estimating equations with robust (sandwich) estimation of SEs to allow for multiple births within a family, using separate models for each brain tissue or structure. The regression was performed first unadjusted, and then adjusted for gestational age at birth, sex, small for gestational age (birth weight ≤ 2 SD below the mean for their age), BPD, and major brain injury (defined as either intraventricular hemorrhage grade 3 or 4, or cystic periventricular leukomalacia as confirmed on neonatal cranial ultrasound). All analyses were corrected for age at the time of the MRI. Results are presented as the mean volumetric difference and 95% CI in cc, as well as the percentage difference calculated as the volumetric difference divided by the mean volume of the brain tissue or structure in the group that did not receive postnatal dexamethasone.

For brain parcel comparisons, a single mixed model was used, combining data from all 20 regions across the 2 hemispheres and using random effects to allow for the correlations between repeated measures on an individual, fitting a separate random effect and a separate error term within each region. Mean differences are presented as the percentage difference relative to the mean size of the region in preterm adolescents who did not receive postnatal dexamethasone (using the average of the left and right measurements). All estimates are adjusted for corrected age at the time of the scan, sex, and hemisphere, allowing the effect of group, sex, and hemisphere to vary by region. We also repeated the analysis adjusting for gestational age at birth, small for gestational age, BPD, major brain injury, and intracranial volume.

The relationships between postnatal dexamethasone total cumulative dose and brain volumes of the various structures and tissues were explored in those who received postnatal dexamethasone using linear regression, fitting a separate model for each volume adjusted for age at MRI scan.

## Results

Of the 225 extremely preterm participants known to be alive at age 8 years, by 18 years 1 had died, 10 could not be found, 26 declined to participate, 3 were living in other states or countries and could not attend, and 5 were too disabled to be assessed for any component of the study, leaving 180 (80%) who were seen at 18 years of age; 162 (90% of those who attended the 18 year follow-up) consented for the MRI, of whom 148 (91%) were considered suitable for analysis. When we compared characteristics of participants who did (n = 148) and did not (n = 77) have analyzable MRI, participants who did not have the MRI were more likely to have major brain injury (19% vs 10%, *P* value = .05) and cerebral palsy confirmed at age 8 years (24% vs 8%, *P* value = .001), and a lower full scale IQ at age 8 years (mean difference [95% CI] of −6.2 [−11.2, −1.1], *P* value = .02).

Participant characteristics for the study population are summarized in Table II. Participants who received postnatal dexamethasone in the newborn period were of lower gestational age and birth weight compared with participants who did not receive postnatal dexamethasone. The proportions who were small for gestational age and with major brain injury in the newborn period, however, were similar in both groups. As expected, there was a higher rate of BPD and cerebral palsy in the group who had received postnatal dexamethasone compared with those who did not.

Volumes of total brain tissue, cortical white matter, thalamus, and all nuclei of the basal ganglia were smaller in participants who received postnatal dexamethasone compared with those who did not, even after adjustment for gestational age at birth, sex, small for gestational age, BPD, and presence of major brain injury, with the exception of total brain tissue volume, which failed to reach statistical significance (Table III). The largest reductions, of approximately 7% in the unadjusted analysis, were seen in the thalamus and basal ganglia. There was little evidence of a difference in cortical gray matter, hippocampus, amygdala, and cerebellar volumes between the groups.

**Figure 1:**
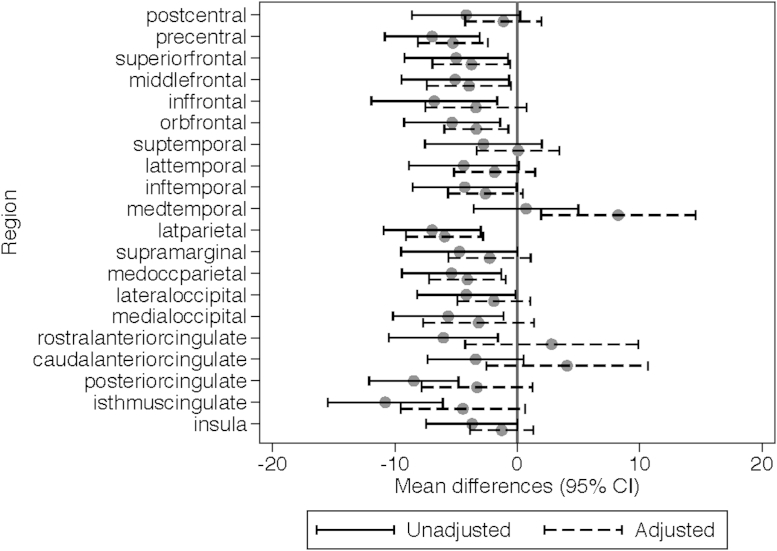
Mean differences (95% CI) in cortical white matter volume in preterm infants who received postnatal dexamethasone compared with preterm infants who did not receive postnatal dexamethasone at 18 years of age. All estimates are adjusted for corrected age at the time of the scan, sex, and hemisphere, allowing the effect of group, sex, and hemisphere to vary by region. Adjusted estimates are also adjusted for gestational age at birth, small for gestational age, bronchopulmonary dysplasia, neonatal brain injury, and intracranial volume.

Given there was evidence of a difference in cortical white matter between groups, we compared white matter regional volumes between those who received postnatal dexamethasone and those who did not (Figure 1). There was evidence that the effect of postnatal dexamethasone varied by region (interaction *P* < .001), with a reduction in brain parcel volumes in those receiving postnatal dexamethasone in most of the white matter regions except the medial temporal region. The evidence for group differences was weaker when adjusted for gestational age at birth, sex, small for gestational age, BPD, presence of major brain injury, and intracranial volume.

**Figure 2:**
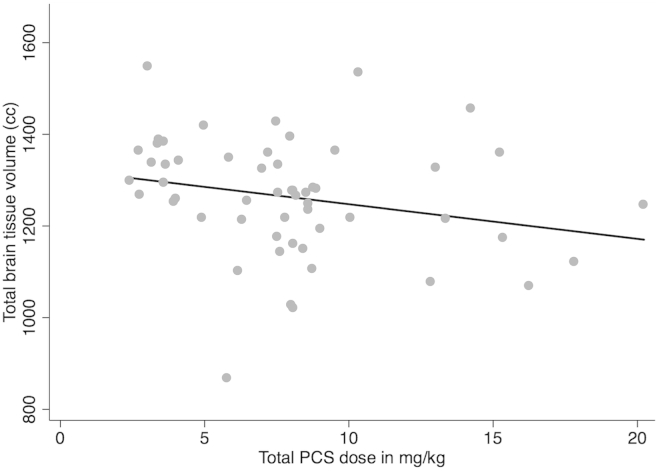
Relationship between total brain tissue volume and cumulative postnatal dexamethasone dose. Regression coefficient −7.7 (95% CI −16.2, 0.8), R^2^ 0.06, *P* value = .08.

There was weak evidence of a relationship between total cumulative dose of postnatal dexamethasone and lower total brain tissue volume [regression coefficient −7.7 (95% CI −16.2, 0.8), R^2^ 0.06, *P* value = .08] (Figure 2) and cortical white matter [regression coefficient −3.2 (95% CI −6.6, 0.2), R^2^ 0.10, *P* value = .06]. There was little evidence of a relationship between total cumulative dose of postnatal dexamethasone and the volumes of other brain tissues or structures (all *P* > .1, data not shown).

## Discussion

Postnatal dexamethasone use to treat or prevent BPD in the newborn period was associated with smaller brain volumes in total brain tissue, cortical white matter, thalamus, and all nuclei of the basal ganglia at age 18 years in a large geographical cohort of extremely preterm adolescents. Smaller white matter volumes associated with postnatal dexamethasone use reflect a global reduction across the majority of the white matter regions. Contrary to previous short-term outcome studies,^8,9,12^ we did not find that postnatal dexamethasone use was associated with reductions in cortical gray matter or cerebellar volumes. We also found that the total dose of postnatal dexamethasone was weakly associated with lower volumes of total brain tissue and cortical white matter in those who received postnatal dexamethasone.

Brain volume alterations associated with postnatal dexamethasone have been reported at term equivalent age, most consistently for total brain tissue, cortical gray matter, and the cerebellum. Previous small studies have reported smaller total brain tissue and cortical gray matter in the order of 10%-30% in a study of 7 preterm infants treated with dexamethasone compared with 11 non-treated preterm infants, and of 9%-35% in 11 preterm infants treated with dexamethasone compared with 30 non-treated preterm infants.^8,9^ The study reporting the largest differences in brain volumes with postnatal dexamethasone use did not adjust for gestational age at time of MRI scan or other potential confounders, and the infants had received a wide range of steroid doses [mean duration (SD) of 28 (22) days, median dose of 0.25 mg/kg/d (range: 0.19-0.90 mg/kg/d)].^8^ A larger study did not find differences in total brain tissue volume at term equivalent age in 123 participants who received an MRI scan at term equivalent age, in groups who received either dexamethasone (n = 17) or hydrocortisone (n = 28) compared with those who received no PCS.^12^ In the current study, we found approximately 3% smaller total brain tissue volume at age 18 years in those who received postnatal dexamethasone, even after adjustment for potential confounders. The fact that the magnitude of difference varies between studies may reflect inherent differences in cohort characteristics, as well as different corticosteriod dosing regimens used. Our findings lend support to the hypothesis that the association between postnatal dexamethasone use and smaller brain volumes, noted as early as term equivalent age, persists into adolescence, with varying effects in the different brain tissues and regions.

Contrary to previous studies, we did not find evidence of smaller cerebellar volumes at age 18 years with postnatal dexamethasone use.^7^ Experimental models have demonstrated deleterious effects on the granule cells of the cerebellum with respect to decreased proliferation and increased apoptosis.^23,24^ Parikh et al^9^ reported a 21% decrease in cerebellar size at term equivalent age in extremely low birth weight (birthweight <1000 g) infants who had received a mean cumulative dose of 2.8 mg/kg of dexamethasone (n = 11) compared with those who received no dexamethasone (n = 30). In another cohort study of 172 babies with a mean gestational age 28 weeks, there were 8% and 10% reductions in cerebellar volumes by 40 weeks postmenstrual age in infants who had received postnatal hydrocortisone (median dose 14 mg/kg) and dexamethasone (median dose 1.2 mg/kg) compared with no PCS use, respectively.^12^ The absence of cerebellar volume difference in adolescence in our study, despite having used higher doses of dexamethasone compared with the 2 studies above, suggests that there may have been ‘catch-up’ growth of the cerebellum in the early years after birth.

In the current study, we found the largest relative volume differences associated with postnatal dexamethasone use in the thalamus and basal ganglia, and to a lesser extent, the cortical white matter. Glucocorticoid receptors have been identified in the posterior thalamus, and corticosteroids have been shown in experimental models to exert effects on these receptors within the thalamus.^25^ Thus, thalamic volume reductions related to postnatal dexamethasone use is plausible. White matter volume reductions related to postnatal dexamethasone have not been reported in previous studies of younger subjects.^8-11,13^ The volume effects of postnatal dexamethasone on different brain tissues or structures compared with studies performed at term equivalent age may reflect vulnerability of specific brain tissues or structures to postnatal dexamethasone and altered trajectory of development that do not become evident until late adolescence. In addition, we were able to demonstrate that the smaller white matter volumes reflected a global change, rather than vulnerability of specific white matter regions, with the exception of larger white matter volumes in the medial temporal region following postnatal dexamethasone use for reasons that are unclear.

There has been much interest in alternative corticosteroids such as hydrocortisone to prevent or treat BPD. At both term equivalent age and also at age 8 years, several studies comparing hydrocortisone-treated preterm infants with preterm infants who received no hydrocortisone reported little evidence of differences in volumes of total brain tissue, cortical gray matter, white matter, deep nuclear gray matter, cerebellum, and hippocampus^10,11,26^; only 1 of these studies was a randomized-controlled trial.^26^ All the studies used hydrocortisone doses that were much lower than the mean 7.9 mg/kg dexamethasone-equivalent dose used in the current study. One study comparing ex-preterm 8-year-olds who received hydrocortisone or not also reported similar mean intelligence.^11^ None of these previous studies were able to confirm that there was any effect of postnatal hydrocortisone on pulmonary outcomes like BPD. In contrast, a larger study documented smaller cerebellar volumes at term equivalent age in infants who had received hydrocortisone compared with those who had not, although the magnitude of difference was less than that observed in infants who had received dexamethasone.^12^ The “hydrocortisone” group in that study did not exclusively receive hydrocortisone as some of the infants also received dexamethasone. There is experimental evidence suggesting that hydrocortisone may be less neurotoxic to the brain than dexamethasone, relating to the fact that hydrocortisone preferentially binds to mineralocorticoid receptors rather than glucocorticoid receptors such as dexamethasone.^27^ Activation of glucocorticoid receptors has been associated with neuronal apoptosis in the hippocampus and cerebellum.^3,4,23,24^ However, the efficacy of hydrocortisone in preventing or treating BPD has not been definitively demonstrated in randomized-controlled trials.^28^

The current study has several strengths. We report findings from a large geographical cohort of extremely preterm infants, prospectively followed up from birth. A high percentage of participants who attended the 18-year follow-up consented for the MRI.

We also acknowledge the limitations. Participants who did not have an MRI were more likely to have major brain injury and cerebral palsy, which may have affected the ability to detect differences in brain tissue volumes between the groups. As the MRI data are cross-sectional, we were not able to infer any developmental changes in brain volume from an earlier age. Brain MRI was not available for neonatal research in 1991-1992, when the study participants were born. It is also important to note that the findings of the current study relate to postnatal dexamethasone prescribing practices dating back 18 years. Since then, lower doses of dexamethasone have been used and proportionally fewer extremely preterm subjects have been treated with dexamethasone in the state of Victoria.^29^ Given the non-random allocation of babies to postnatal dexamethasone, we describe an association, albeit a robust one, between postnatal dexamethasone and reduced brain growth, rather than infer causality.

Postnatal dexamethasone use in the newborn period in extremely preterm infants is associated with brain volume reductions in adolescence, particularly total brain tissue, cortical white matter, thalamus, and basal ganglia. This highlights potential vulnerability of the brain to dexamethasone that persist into adolescence. Although there is a suggestion that alternative PCS like hydrocortisone is not associated with alterations in brain size, studies to date are relatively short-term with limited information on functional neurodevelopmental correlates. Future studies should include clarification of long-term brain alterations in association with functional outcomes of preterm infants using alternative PCS or lower dose regimens of dexamethasone.

## Figures and Tables

**Table I:** Twenty brain parcellated regions derived from the 34 regions generated from the automated Freesurfer program^1^

Brain lobes	Freesurfer-generated brain regions	Brain region labels used for this study
Frontal	Postcentral gyrus	Postcentral
	Precentral gyrus	Precentral
	Superior frontal gyrus	Superior frontal
	Middle frontal gyrus (caudal + rostral division) + frontal pole	Middle frontal
	Pars opercularis + pars triangularis	Inferior frontal
	Pars orbitalis + orbitofrontal cortex (lateral + medial division)	Orbital frontal
Temporal	Superior temporal gyrus + transverse temporal cortex	Superior temporal
	Middle temporal gyrus + banks of the superior temporal sulcus	Lateral temporal
	Inferior temporal gyrus + fusiform gyrus	Inferior temporal
	Temporal pole + entorhinal cortex + Parahippocampal gyrus	Medial temporal
Parietal	Inferior parietal cortex + superior parietal cortex	Lateral parietal
	Supramarginal gyrus	Supramarginal
	Cuneus cortex + precuneus cortex + Paracentral Lobule	Medial occipital parietal
Occipital	Lateral occipital cortex	Lateral occipital
	Lingual gyrus + pericalcarine cortex	Medial occipital
Other	Cingulate cortex - rostral anterior division	Rostral anterior cingulate
	Cingulate cortex - caudal anterior division	Caudal anterior cingulate
	Cingulate cortex - posterior division	Posterior cingulate
	Cingulate cortex - isthmus division	Isthmus
	Insula	Insula

**Table II:** Participant characteristics

	Received PCS (n = 55)	No PCS (n = 93)
Multiple births	22 (23)	13 (24)
Gestational age at birth in wk, mean (SD)	25.3 (1.1)	26.2 (0.9)
Birthweight in g, mean (SD)	785 (145)	963 (160)
Birthweight ≤2 SD	3 (5)	1 (1)
Male	31 (56)	39 (42)
Antenatal corticosteroids	40 (73)	62 (67)
Major brain injury∗	5 (9)	10 (11)
BPD†	36 (65)	21 (23)
Duration of PCS treatment in d, median (IQR)	27 (17-39)	-
Total cumulative PCS dose in mg/kg, mean (SD)	7.9 (4.0)	-
Day of life at PCS commencement, mean (SD)	23.9 (7.7)	-
Corrected age at PCS commencement in wk, mean (SD)	29.2 (1.5)	-
Cerebral palsy‡	6 (11)	5 (5)
IQ,§ mean (SD)	90.9 (16.0)	98.5 (15.2)

Data are n (%) unless otherwise specified.

∗ Defined as intraventricular hemorrhage grade 3 or 4 and/or cystic periventricular leukomalacia.

† Defined as oxygen requirement at age 36 wk.

‡ Confirmed at age 8 years.

§ Assessed at age 18 years using the Wechsler Abbreviated Scale of Intelligence; data available for 54 participants in the PCS group.

**Table III:** Comparison of brain volumes in participants who received postnatal dexamethasone compared with those who did not

Brain tissue or structure	Brain volume (cc) mean (SD)		Mean difference (95% CI)		*P* value	Adjusted mean difference∗ (95% CI)		Adjusted *P* value∗
	Postnatal dexamethasone (n = 55)	No postnatal dexamethasone (n = 93)	Volume (cc)	Volume (%)†		Volume (cc)	Volume (%)†	
Total brain tissue	1262.9 (126.9)	1305.7 (126.6)	−47.0 (−90.7, −3.3)	−3.6 (−7.0, −0.3)	.04	−35.6 (−74.5, 3.2)	−2.7 (−5.7, 0.2)	.07
Cortical gray matter	493.6 (48.3)	500.7 (49.9)	−10.1 (−26.9, 7.0)	−2.0 (−5.4, 1.4)	.25	−9.6 (−24.0, 4.8)	−1.9 (−4.8, 1.0)	.19
Cortical white matter	422.9 (51.8)	447.0 (54.1)	−23.4 (−41.4, −5.4)	−5.2 (−9.3, −1.2)	.01	−19.4 (−37.5, −1.4)	−4.4 (−8.4, −0.3)	.03
Thalamus	13.6 (1.5)	14.6 (1.6)	−1.1 (−1.6, −0.6)	−7.5 (−11.0, −4.1)	<.001	−0.9 (−1.4, −0.4)	−6.0 (−9.6, −2.4)	.001
Basal ganglia	21.0 (2.5)	22.6 (2.5)	−1.7 (−2.5, −0.8)	−7.5 (−11.1, −3.5)	<.001	−1.3 (−2.1, −0.5)	−5.7 (−9.2, −2.2)	.002
Caudate	7.1 (1.1)	7.7 (1.0)	−0.6 (−1.0, −0.2)	−7.8 (−13.0, −2.6)	.001	−0.4 (−0.8, −0.1)	−5.5 (−10.0, −0.9)	.02
Putamen	11.0 (1.1)	11.6 (1.4)	−0.7 (−1.1, −0.3)	−6.0 (−9.5, −2.6)	.001	−0.6 (−1.0, −0.2)	−4.8 (−8.4, −1.3)	.007
Pallidum	2.9 (0.4)	3.3 (0.5)	−0.3 (−0.5, −0.2)	−9.1 (−15.2, −6.1)	<.001	−0.3 (−0.5, −0.2)	−9.4 (−13.6, −5.2)	<.001
Cerebellum	140.8 (19.5)	144.8 (16.6)	−4.3 (−10.5, 2.0)	−3.0 (−7.3, 1.4)	.18	−1.3 (−7.1, 4.4)	−0.9 (−4.9, 3.0)	.65
Hippocampus	8.0 (0.7)	8.1 (0.8)	−0.1 (−0.4, 0.1)	−1.2 (−4.9, 1.2)	.35	−0.2 (−0.4, 0.1)	−2.0 (−4.9, 1.1)	.21
Amygdala	3.3 (0.3)	3.4 (0.3)	−0.1 (−0.2, 0.1)	−2.9 (−5.9, 1.2)	.20	−0.1 (−0.2, 0.04)	−2.1 (−5.6, 1.2)	.21

All analysis were adjusted for age at MRI.

∗ Also adjusted for gestational age at birth, sex, small for gestational age, bronchopulmonary dysplasia, and major brain injury.

† Volume difference expressed as a percentage of the mean volume in the participants who did not receive postnatal dexamethasone for the particular brain tissue or structure.

## Appendix

Additional members of the Victorian Infant Collaborative Study Group include:

Premature Infant Follow-up Program at the Royal Women's Hospital, Melbourne, Australia: Catherine Callanan, RN, Esther Hutchinson, BSc (Hons), Marion McDonald, RN, Karissa Searle, BSc; Premature Infant Follow-up Program at the Royal Women's Hospital and Obstetrics and Gynecology, the University of Melbourne, Melbourne, Australia: Cinzia R. De Luca, BSc, PhD, Carly Molloy, PhD, Michelle Wilson-Ching, PhD; Premature Infant Follow-up Program at the Royal Women's Hospital and Mercy Hospital for Women, Melbourne, Australia: Elaine Kelly, MA; Monash Medical Centre, Melbourne, Australia: Elizabeth Carse, FRACP, Margaret P. Charlton, M Ed Psych, Marie Hayes, RN; Mercy Hospital for Women, Melbourne, Australia: Gillian Opie, FRACP, Andrew Watkins, FRACP, Amanda Williamson, Heather Woods, RN; and Royal Children's Hospital, Melbourne, Australia: Colin Robertson, MD, FRACP (funded by Australian National Health and Medical Research Council [491246].

## References

[1] Halliday H.L., Ehrenkranz R.A., Doyle L.W. (2010). Early (<8 days) postnatal corticosteroids for preventing chronic lung disease in preterm infants. Cochrane Database Syst Rev.

[2] Doyle L.W., Ehrenkranz R.A., Halliday H.L. (2010). Dexamethasone treatment in the first week of life for preventing bronchopulmonary dysplasia in preterm infants: a systematic review. Neonatology.

[3] Tombaugh G.C., Yang S.H., Swanson R.A., Sapolsky R.M. (1992). Glucocorticoids exacerbate hypoxic and hypoglycemic hippocampal injury in vitro: biochemical correlates and a role for astrocytes. J Neurochem.

[4] Uno H., Eisele S., Sakai A., Shelton S., Baker E., DeJesus O. (1994). Neurotoxicity of glucocorticoids in the primate brain. Hormones Behav.

[5] Benesová O., Pavlík A. (1989). Perinatal treatment with glucocorticoids and the risk of maldevelopment of the brain. Neuropharmacology.

[6] Weichsel M.E. (1977). The therapeutic use of glucocorticoid hormones in the perinatal period. Potential neurological hazards. Ann Neurol.

[7] Pavlík A., Buresová M. (1984). The neonatal cerebellum: the highest level of glucocorticoid receptors in the brain. Brain Res.

[8] Murphy B.P., Inder T.E., Huppi P.S., Warfield S., Zientara G.P., Kikinis R. (2001). Impaired cerebral cortical gray matter growth after treatment with dexamethasone for neonatal chronic lung disease. Pediatrics.

[9] Parikh N.A., Lasky R.E., Kennedy K.A., Moya F.R., Hochhauser L., Romo S. (2007). Postnatal dexamethasone therapy and cerebral tissue volumes in extremely low birth weight infants. Pediatrics.

[10] Benders M.J.N.L., Groenendaal F., van Bel F., Ha Vinh R., Dubois J., Lazeyras F. (2009). Brain development of the preterm neonate after neonatal hydrocortisone treatment for chronic lung disease. Pediatr Res.

[11] Lodygensky G.A., Rademaker K., Zimine S., Gex-Fabry M., Lieftink A.F., Lazeyras F. (2005). Structural and functional brain development after hydrocortisone treatment for neonatal chronic lung disease. Pediatrics.

[12] Tam EW, Chau V, Ferriero DM, Barkovich AJ, Poskitt KJ, Studholme C, et al. Preterm cerebellar growth impairment after postnatal exposure to glucocorticoids. Sci Transl Med 2011;3:105ra. 10.1126/scitranslmed.3002884 PMC3682111 22013125

[13] Kersbergen K.J., de Vries L.S., van Kooij B.J., Isgum I., Rademaker K.J., van Bel F. (2013). Hydrocortisone treatment for bronchopulmonary dysplasia and brain volumes in preterm infants. J Pediatr.

[14] Thompson D.K., Wood S.J., Doyle L.W., Warfield S.K., Lodygensky G.A., Anderson P.J. (2008). Neonate hippocampal volumes: prematurity, perinatal predictors, and 2-year outcome. Ann Neurol.

[15] The Victorian Infant Collaborative Study Group (1997). Outcome at 2 years of children 23-27 weeks' gestation born in Victoria in 1991-1992. J Paediatr Child Health.

[16] Anderson P., Doyle L.W., Victorian Infant Collaborative Study Group (2003). Neurobehavioral outcomes of school-age children born extremely low birth weight or very preterm in the 1990s. JAMA.

[17] Doyle L.W. (2001). Outcome at 5 years of age of children 23 to 27 weeks' gestation: refining the prognosis. Pediatrics.

[18] Doyle L.W., Anderson P.J. (2005). Improved neurosensory outcome at 8 years of age of extremely low birth weight children born in Victoria over three distinct eras. Arch Dis Child Fetal Neonatal Ed.

[19] Dale A.M., Fischl B., Sereno M.I. (1999). Cortical surface-based analysis: I. Segmentation and surface reconstruction. NeuroImage.

[20] Fischl B., Salat D.H., Busa E., Albert M., Dieterich M., Haselgrove C. (2002). Whole brain segmentation: automated labeling of neuroanatomical structures in the human brain. Neuron.

[21] Desikan R.S., Segonne F., Fischl B., Quinn B.T., Dickerson B.C., Blacker D. (2006). An automated labeling system for subdividing the human cerebral cortex on MRI scans into gyral based regions of interest. NeuroImage.

[22] Salat D.H., Greve D.N., Pacheco J.L., Quinn B.T., Helmer K.G., Buckner R.L. (2009). Regional white matter volume differences in nondemented aging and Alzheimer's disease. NeuroImage.

[23] Noguchi K.K., Walls K.C., Wozniak D.F., Olney J.W., Roth K.A., Farber N.B. (2008). Acute neonatal glucocorticoid exposure produces selective and rapid cerebellar neural progenitor cell apoptotic death. Cell Death Differ.

[24] Bohn M.C., Lauder J.M. (1980). Cerebellar granule cell genesis in the hydrocortisone-treated rats. Dev Neurosci.

[25] Jaferi A., Bhatnagar S. (2006). Corticosterone can act at the posterior paraventricular thalamus to inhibit hypothalamic-pituitary-adrenal activity in animals that habituate to repeated stress. Endocrinology.

[26] Parikh N.A., Kennedy K.A., Lasky R.E., McDavid G.E., Tyson J.E. (2013). Pilot randomized trial of hydrocortisone in ventilator-dependent extremely preterm infants: effects on regional brain volumes. J Pediatr.

[27] de Kloet E.R., Vreugdenhil E., Oitzl M.S., Joëls M. (1998). Brain corticosteroid receptor balance in health and disease. Endocrine Rev.

[28] Doyle L.W., Ehrenkranz R.A., Halliday H.L. (2010). Postnatal hydrocortisone for preventing or treating bronchopulmonary dysplasia in preterm infants: a systematic review. Neonatology.

[29] Cheong J.L., Anderson P., Roberts G., Duff J., Doyle L.W., Victorian Infant Collaborative Study Group (2013). Postnatal corticosteroids and neurodevelopmental outcomes in extremely low birth weight or extremely preterm infants: 15-year experience in Victoria, Australia. Arch Dis Child Fetal Neonatal Ed.

